# Clonal hematopoiesis in people with advanced HIV and associated inflammatory syndromes

**DOI:** 10.1172/jci.insight.174783

**Published:** 2024-04-02

**Authors:** Joseph M. Rocco, Yifan Zhou, Nicholas S. Liu, Elizabeth Laidlaw, Frances Galindo, Megan V. Anderson, Adam Rupert, Silvia L. Lage, Ana M. Ortega-Villa, Shiqin Yu, Andrea Lisco, Maura Manion, George S. Vassiliou, Cynthia E. Dunbar, Irini Sereti

**Affiliations:** 1 National Institute of Allergy and Infectious Diseases, and; 2 Translational Stem Cell Biology Branch, National Heart, Lung and Blood Institute, NIH, Bethesda, Maryland, USA.; 3 Wellcome-MRC Cambridge Stem Cell Institute, University of Cambridge, Puddicombe Way, Cambridge, United Kingdom.; 4 Wellcome Sanger Institute, Wellcome Genome Campus, Hinxton, Cambridge, United Kingdom.; 5 Department of Biology, Brown University, Providence, Rhode Island, USA.; 6 Leidos Biomedical Research, Inc, Frederick, Maryland, USA.; 7 Department of Haematology, Cambridge University Hospitals NHS Foundation Trust (CUH), Cambridge, United Kingdom.

**Keywords:** AIDS/HIV, Aging, Clonal selection, Macrophages, T cells

## Abstract

People with HIV (PWH) have a higher age-adjusted mortality due to chronic immune activation and age-related comorbidities. PWH also have higher rates of clonal hematopoiesis (CH) than age-matched non-HIV cohorts; however, risk factors influencing the development and expansion of CH in PWH remain incompletely explored. We investigated the relationship between CH, immune biomarkers, and HIV-associated risk factors (CD4^+^ and CD8^+^ T cells, nadir CD4^+^ count, opportunistic infections [OIs], and immune reconstitution inflammatory syndrome [IRIS]) in a diverse cohort of 197 PWH with median age of 42 years, using a 56-gene panel. Seventy-nine percent had a CD4^+^ nadir below 200 cells/μL, 58.9% had prior OIs, and 34.5% had a history of IRIS. The prevalence of CH was high (27.4%), even in younger individuals, and CD8^+^ T cells and nadir CD4^+^ counts strongly associated with CH after controlling for age. A history of IRIS was associated with CH in a subgroup analysis of patients 35 years of age and older. Inflammatory biomarkers were higher in CH carriers compared with noncarriers, supporting a dysregulated immune state. These findings suggest PWH with low nadir CD4^+^ and/or inflammatory complications may be at high risk of CH regardless of age and represent a high-risk group that could benefit from risk reduction and potentially targeted immunomodulation.

## Abstract 2

People with advanced human immunodeficiency virus with low CD4 T-cell counts, opportunistic infections, or associated inflammatory syndromes are at increased risk of clonal hematopoiesis.

## Introduction

The advent of antiretroviral therapy (ART) has dramatically improved the life expectancy of people with HIV (PWH). Rapid initiation of ART further improves mortality; however, advanced-stage presentations of HIV with blood CD4^+^ T cell counts below 200 cells/μL continue to occur, including in middle-high income areas, despite widespread availability of ART (1–4). Even with suppression of detectable plasma HIV virus and CD4^+^ T cell recovery, PWH have higher age-adjusted mortality and increased rates of serious non-AIDS adverse events, including malignancies and cardiovascular disease (CVD), compared with age-matched cohorts without HIV (5–7). This heightened risk of age-associated diseases in PWH is associated with elevated inflammatory biomarkers and myeloid cell immune activation (4). A major risk factor for chronic inflammation in PWH is the depth of nadir blood CD4^+^ T cell frequency prior to ART initiation (8). Mechanisms driving this chronic inflammation in well-controlled HIV remain unknown, limiting the investigation of targeted therapeutics.

Clonal hematopoiesis (CH) is an age-related phenomenon driven by the acquisition of somatic mutations in leukemia-associated genes in a hematopoietic stem cell (HSC), resulting in clonal expansion of the mutant HSC and its progeny, particularly myeloid lineage cells (9–13). Mutations linked to CH occur predominately in the epigenetic regulator genes *DNMT3A*, *TET2*, and *ASXL1*. Myeloid lineage cells with these mutations have a proinflammatory phenotype (14). Those with CH have increased all-cause mortality driven by higher rates of CVD attributed to increases in systemic inflammation, in addition to a greater risk of hematologic malignancies and other diseases of aging (15). Chronic inflammation can accelerate clonal expansion and increase the prevalence of clinically relevant levels of mutant cells, likely due to increased inflammatory cytokine receptor expression in clonal hematopoietic stem and progenitor cells (HSPCs) and resistance to inflammatory exhaustion in comparison with nonmutated HSPCs (16–19).

Untreated HIV results in a chronic inflammatory state in addition to progressive immunodeficiency. Some people with advanced HIV and underlying opportunistic infections (OIs) develop a paradoxical hyperinflammatory reaction termed immune reconstitution inflammatory syndrome (IRIS) after starting ART (20, 21). In patients who develop severe IRIS in the setting of mycobacterial infections, this inflammatory state can resemble secondary hemophagocytic lymphohistiocytosis (HLH), another hyperinflammatory syndrome requiring prolonged immune suppression in this population (22).

It is well established in other disease models that inflammation can accelerate CH development (16, 23, 24). Due to the known link between CH and hyperinflammatory pathways, we hypothesized that CH could accelerate in PWH, and lead to higher rates of chronic inflammation and comorbidities even after HIV is well controlled. Recent studies identified an increased prevalence of CH in PWH, highest in those with lower nadir CD4^+^ counts (25–28). However, published studies included fewer patients with a nadir CD4^+^ count below 200 cells/μL, and the impact of CH in HIV cohorts with prior AIDS and/or IRIS remains unexplored (29).

We evaluated CH in a cohort of PWH with a broad range of nadir CD4^+^ counts and a history of diverse OIs, including a subset that developed IRIS, to further investigate the relationship between CH and HIV-associated inflammatory complications. We analyzed the associations between CH, CD4^+^ and CD8^+^ counts, inflammatory syndromes, and biomarkers of inflammation. We also evaluated CH before and after ART in a subset of individuals with OIs and examined the levels of CH mutations in various cell lineages in a subgroup of our overall cohort. Identifying PWH at high risk of long-term, serious non-AIDS adverse events is important, as this population may benefit from more aggressive risk reduction and could be candidates for targeted immune modulation to decrease their basal inflammatory state.

## Results

### Clinical characteristics.

The 197 PWH included in this study were enrolled and followed longitudinally at the NIH between 2006 and 2022. All individuals had successful suppression of plasma HIV viral load below the limit of detection. Peripheral blood mononuclear cells (PBMCs) were collected from individuals with a suppressed HIV viral load after ART, referred to as the Follow-up time point. No individuals were on corticosteroids or had untreated OIs at the time of this sample collection. Forty-one individuals (20.8%) had a CD4^+^ nadir of greater than 200 cells/μL, whereas 156 (79.2%) had a CD4^+^ nadir of less than 200 cells/μL. Of these 156 PWH, 68 had a history of IRIS. A subset of 82 patients with mycobacterial infections and/or severe IRIS had PBMC samples available also from before ART initiation or during the IRIS event and these samples were evaluated for CH, referred to as the Acute time point.

Median age at the Follow-up sample time point was 42 years (interquartile range [IQR]: 36–52), with more men (*n* = 145) than women (*n* = 52, 26.4%) (Table 1). Individuals with a CD4^+^ nadir of less than 200, including the subgroup with IRIS, were significantly younger, with a median age of 40 years (IQR: 35–47) compared with those with CD4^+^ nadir of greater than 200, with a median age of 55 years (IQR: 48–59). The full cohort was ethnically and racially diverse, with African/African American (*n* = 117), non-White/Hispanic (*n* = 41), and White/non-Hispanic (*n* = 20) persons. There were 83 participants (42.1%) who reported active or prior history of tobacco use. A history of OIs from intracellular pathogens (mycobacteria, histoplasmosis, cryptococcosis, toxoplasmosis) and/or chronic viral infections such as Kaposi’s sarcoma herpesvirus (KSHV) were common, with 116 (58.9%) individuals having a history of at least one of these OIs. There were also 7 (3.6%) PWH with 2 prior OIs, and 1 (0.5%) with 3. The most common AIDS-defining diseases in the cohort were disseminated mycobacterial infection (*n* = 76, 38.6%), KSHV-associated disorders (*n* = 20, 10.2%), histoplasmosis (*n* = 12, 6.1%), cryptococcosis (*n* = 10, 5.1%), lymphoma (*n* = 8, 4.1%), and toxoplasmosis (*n* = 7, 3.6%). Of the 41 PWH with a CD4^+^ nadir of greater than 200, 18 (5.1%) met criteria for long-term nonprogressor/elite controller status (LTNP/EC) (30).

### CH carrier prevalence in PWH.

**Figure 1:**
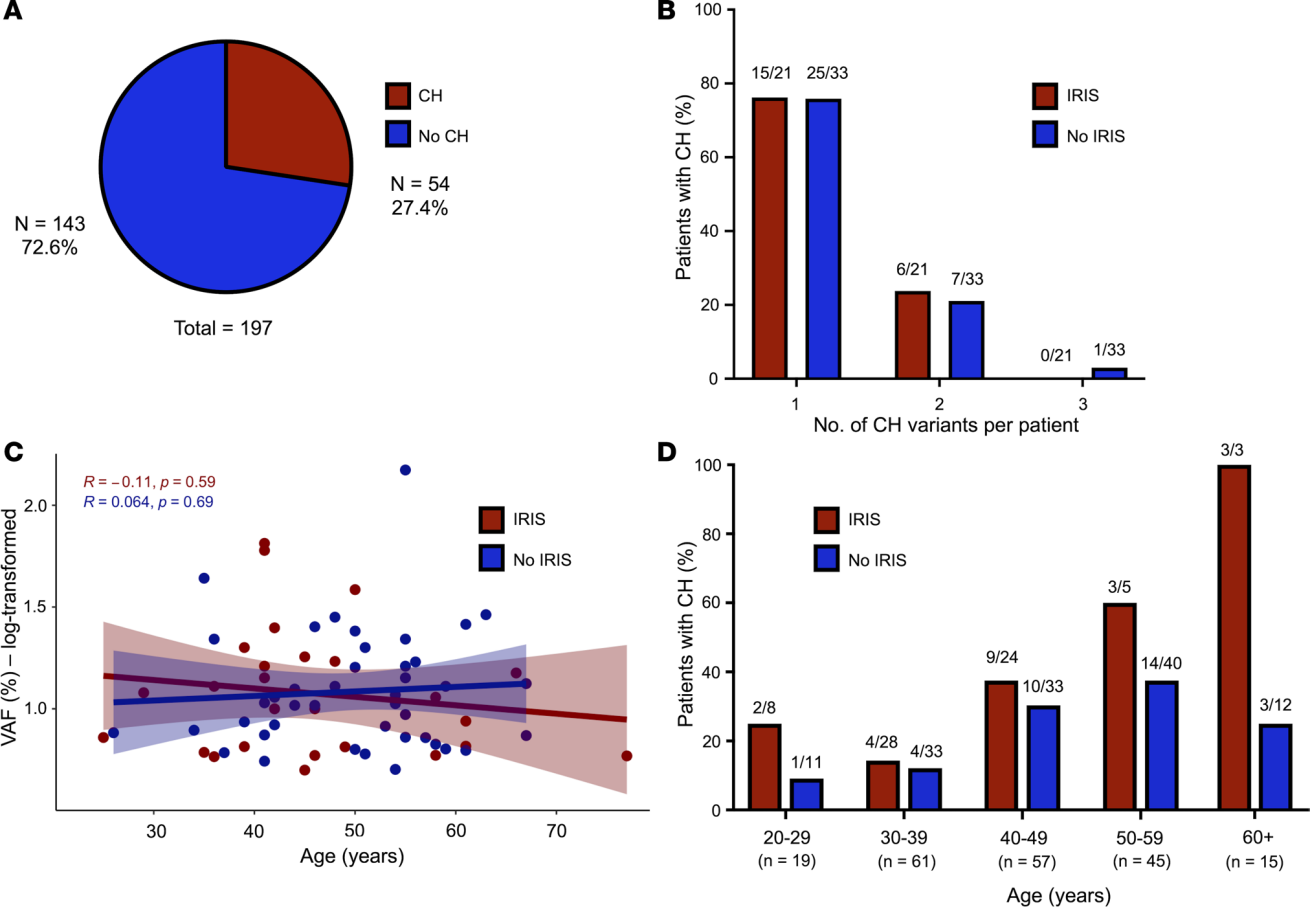
Characteristics of clonal hematopoiesis in people with HIV analyzed according to a history of IRIS. (**A**) Proportion of participants with any clonal hematopoiesis (CH) mutation. (**B**) Number of CH variants identified per individual stratified by history of immune reconstitution inflammatory syndrome (IRIS). (**C**) Variant allele frequency (VAF; log transformed) over increasing age stratified by history of IRIS. Median values are represented by linear regression lines, and the shaded area represents the upper and lower 95% CIs. *R* represents Spearman’s correlation coefficient from each group with *P* value. (**D**) Proportion of individuals with any CH mutation by age group and history of IRIS.

A total of 69 unique protein-changing CH variants were identified in 54 of the 197 PWH (27.4%) at the Follow-up time point, with 14 individuals (7.1%) carrying multiple CH variants (Figure 1, A and B), using an error-corrected sequencing platform targeting 56 commonly mutated CH genes able to detect somatic mutations down to a variant allele frequency (VAF) of 0.5% (31). CH variants were identified most frequently in *DNMT3A* (46.4%) and *TET2* (18.8%), and missense mutations were most common (Supplemental Figure 1; supplemental material available online with this article; https://doi.org/10.1172/jci.insight.174783DS1). Variants were also identified in *STAT3*, *TP53*, *ASXL1*, *CBL*, *PPM1D*, and *KDM6A*. The VAF of CH variants ranged from 0.5% to 14.9%, with a median of 1.07%. As expected, CH prevalence in this cohort increased with age. Of interest, CH prevalence was consistently higher in patients with prior IRIS (IRIS 30.9% vs. no IRIS 24.8%), although this was not statistically significant (χ^2^ test, *P* = 0.36) (Figure 1, C and D). Notably, CH variants were identified in 11 of 80 PWH (13.8%) younger than 40 years of age, including 3 of 19 individuals (15.8%) 20 to 29 years old. A logistic regression model including previously described factors known to impact CH risk (age, sex, race/ethnicity, tobacco use, and history of lymphoma and/or chemotherapy) identified only age as a significant association of CH, with an odds ratio (OR) of 1.05 (95% CI 1.02–1.09) per year.

### Factors associated with CH in PWH.

We next explored HIV-associated variables that could impact the risk of CH development and expansion, including current CD4^+^ and CD8^+^ T cell counts, CD4^+^/CD8^+^ ratio, nadir CD4^+^ count, prior mycobacterial infection or other chronic OIs (KSHV, histoplasmosis, cryptococcosis, toxoplasmosis), history of inflammatory complications including IRIS with or without HLH, and LTNP/EC status (Supplemental Table 1). Nadir CD4^+^ was evaluated both as a continuous and a categorical (<200 cells/μL) variable. Logistic regression was performed independently for each of the aforementioned variables, while controlling for known cofactors associated with CH (age, sex, race/ethnicity, tobacco use, and history of lymphoma and/or chemotherapy).

**Figure 2:**
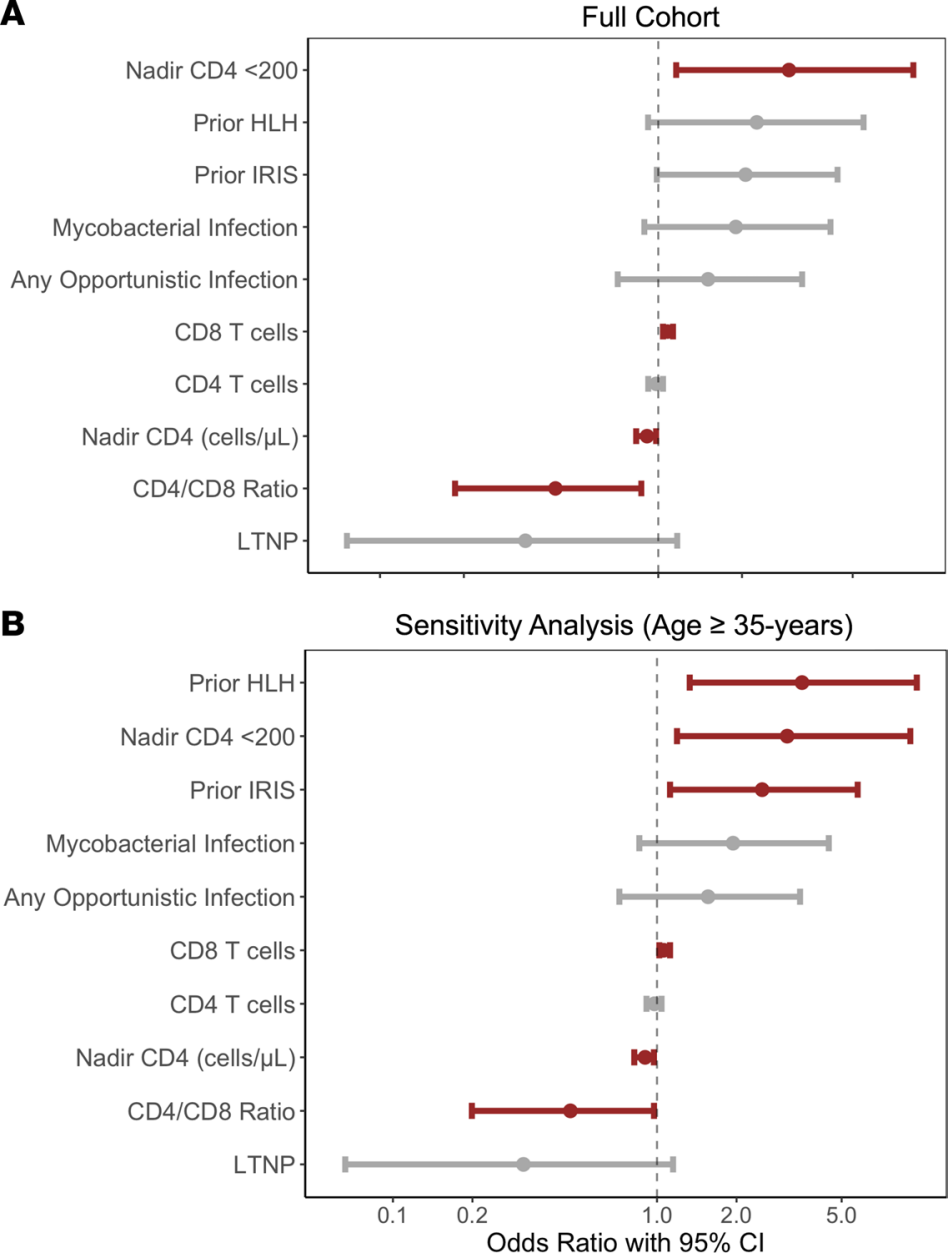
Adjusted logistic regression with 95% CIs of key variables and their impact on risk of clonal hematopoiesis in people with HIV. (**A**) Each HIV-associated variable was independently evaluated with logistic regression models while controlling for known factors that could impact clonal hematopoiesis (CH) risk (age, sex, race/ethnicity, smoking history, lymphoma/chemotherapy history) (*n* = 197). Odds ratios (OR) with 95% CIs are depicted. CD4^+^ and CD8^+^ T cell counts are presented as an OR per 50 cells/μL change. CD4^+^ T cell nadir was evaluated as a categorical variable (CD4^+^ T cell nadir <200 cells/μL) and a continuous variable (per 50 cells/μL change). (**B**) Sensitivity analysis was performed due to enrichment of younger individuals with a history of IRIS (*n* = 158). Logistic regression models evaluating the same key variables and controlling for the same cofactors were repeated, including only individuals ≥35 years of age. IRIS, immune reconstitution inflammatory syndrome; HLH, hemophagocytic lymphohistiocytosis.

Using this logistic regression model, we found that current CD8^+^ T cell counts and nadir CD4^+^ counts, both as categorical and continuous variables, showed the strongest association with CH after controlling for the aforementioned CH risk factors (Figure 2A). Those with a nadir CD4^+^ count of less than 200 cells/μL had an OR of 3.03 (95% CI 1.19–8.46). For every increase in CD8^+^ T cells of 50 cells/μL there was an increased risk of CH by an OR of 1.08 (95% CI 1.04–1.13), and for each increase in nadir CD4^+^ count of 50 cells/μL, there was a decrease in the OR for CH of 0.91 (95% CI 0.83–0.98). A higher CD4^+^/CD8^+^ ratio also associated with a lower CH risk (OR 0.43, 95% CI 0.19–0.87). History of IRIS with or without HLH showed a strong trend toward greater CH, with an OR of 2.06 (95% CI 0.99–4.41) and OR of 2.29 (95% CI 0.91–5.71), respectively. Prior mycobacterial infection also trended toward an increased CH risk. LTNP/EC status was associated with a trend toward a lower rate of CH, with an OR of 0.33 (95% CI 0.07–1.14). Using a forward selection multivariate logistic regression model selecting significant variables from the prior models while also including the original baseline cofactors, only increasing age (OR 1.07, 95% CI 1.03–1.11), CD8^+^ T cells (OR 1.08, 95% CI 1.03–1.13), and nadir CD4^+^ less than 200 (OR 3.03, 95% CI 1.19–8.46) remained statistically associated with a greater risk of CH.

This cohort of PWH was skewed toward younger patients with a history of IRIS with or without HLH, with 36 of 68 (53%) of patients with IRIS with or without HLH being less than 40 years old compared with only 44 of 129 (34.1%) without IRIS. Although we attempted to limit this confounding by performing multivariate regression analyses, we also performed a sensitivity analysis, excluding individuals younger than 35 years old (*n* = 158 after excluding the youngest quartile from the cohort) in order to preserve power while evaluating for associations in key covariates in an older population with a greater risk of CH. Logistic regression models were repeated, controlling for the same 5 cofactors, and this analysis identified prior HLH as the strongest association with CH, with an OR of 3.42 (95% CI 1.26–9.47) (Figure 2B). Nadir CD4^+^ less than 200, CD8^+^ T cells, and history of IRIS were also linked to CH with an OR of 3.18 (95% CI 1.22–9.29), OR of 1.06 (95% CI 1.02–1.11), and OR of 2.34 (95% CI 1.06–5.29), respectively. For every 50 cells/μL increase in nadir CD4^+^, there was a reduction in CH risk (OR 0.98, 95% CI 0.96–0.99).

### Genes with somatic CH variants.

When considering all time points tested, a total of 100 nonsynonymous coding driver CH variants were identified in 54 PWH (27.4%), including those with variants detected at either or both the Acute and Follow-up time points (Supplemental Table 2). *DNMT3A* and *TET2* were the most commonly affected genes. These genes are the most frequently impacted in other cohorts, and both have been previously linked with an enhanced inflammatory state (18, 19, 23). *DNMT3A* variants were the most frequently found in those with a history of IRIS with or without HLH, whereas *TET2* was more common in PWH without inflammatory complications when compared with IRIS patients. Logistic regression analysis for associations of *DNMT3A-*CH (controlling for the same 5 cofactors as for CH risk overall) identified prior IRIS and CD8^+^ T cells as the only significant association (OR 2.53 [95% CI 1.03–6.33] and OR 1.07 per increase of 50 cells/μL [95% CI 1.02–1.12], respectively) (Supplemental Figure 2). In those PWH 35 years and older, a history of IRIS with or without HLH associated with *DNMT3A-*CH variants with ORs of 2.97 (95% CI 1.16–7.85) and 3.51 (95% CI 1.12–10.8), respectively. There were no significant associations with HIV-specific risk factors and CH variants in *TET2*, although power was limited for this comparison due to the sample size (*n* = 13).

To analyze for CH mutations in various blood lineages of interest, we randomly selected 7 patients with available PBMCs at the Follow-up time point who carried *TET2* or *DNMT3A* CH mutations. PBMCs were sorted into CD4^+^ T cells, CD8^+^ T cells, and monocytes. Targeted deep sequencing was used to evaluate the mutations identified in total PBMCs in each of the subsets. *DNMT3A* variants had highly variable VAFs in both T cells and monocytes, while *TET2* mutations were found at much higher VAFs in monocytes compared with T cells in 2 of 3 patients tested, demonstrating a myeloid skewing similar to that reported in a non–HIV-cohort study (Supplemental Figure 3) (32).

### Impact of CH on inflammatory biomarkers.

**Figure 3:**
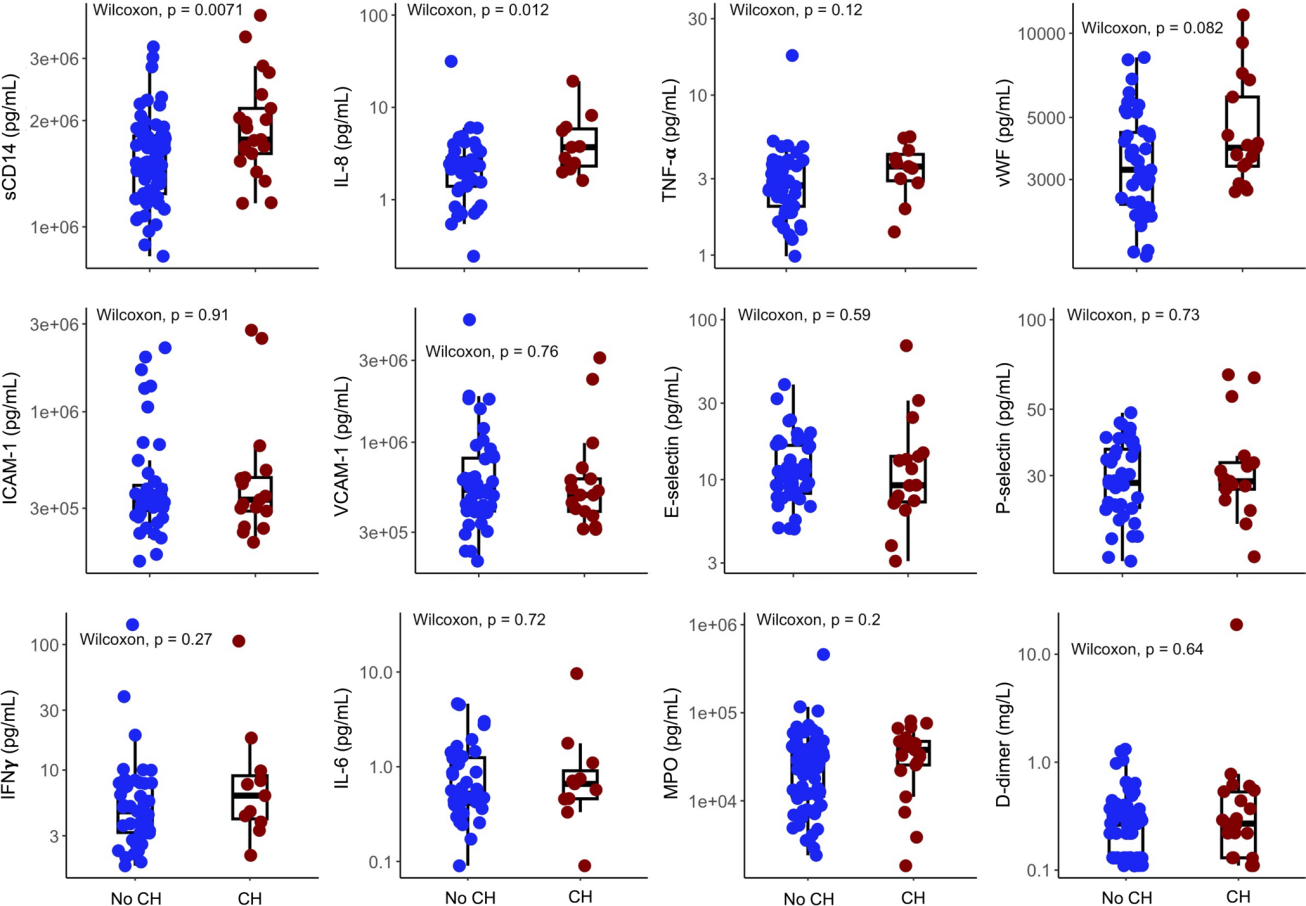
Comparison of biomarkers in people with HIV with or without clonal hematopoiesis at the follow-up time point. Available biomarkers were compared by clonal hematopoiesis (CH) status (*n* = 89). Data are presented as box-and-whisker plots with medians (lines within boxes) and IQRs (box bounds). Groups were compared by Wilcoxon’s rank sum test and resultant *P* values are shown. sCD14, soluble CD14; IL-8, interleukin-8; TNF-α, tumor necrosis factor α; vWF, von Willebrand factor; ICAM-1, intercellular adhesion molecule 1; VCAM-1, vascular cell adhesion molecule 1; IFN-γ, interferon γ; MPO,myeloperoxidase.

Inflammatory and endothelial biomarkers were previously reported from the Follow-up time point used in this study in a subset of 89 PWH included in this current cohort. No differences were found in these markers between those with a history of IRIS and those without (33). We reanalyzed these biomarkers, comparing those with CH (*n* = 21, 23.6%) to those without CH (*n* = 68, 76.4%). Soluble CD14 (sCD14), a marker of increased myeloid cell activation, was significantly increased in those with CH (Figure 3). IL-8, an inflammatory chemokine, was also higher, and there was a trend toward more elevated levels of von Willebrand factor (vWF) in CH carriers. No differences were found in other endothelial activation markers. Trends for higher levels of IFN-γ, TNF-α, and myeloperoxidase (MPO) were found in those with CH compared with those without. No difference was found between absolute monocyte and lymphocyte counts at this time point (Supplemental Figure 4).

### Prevalence of CH at the start of ART and evolution over time.

**Figure 4:**
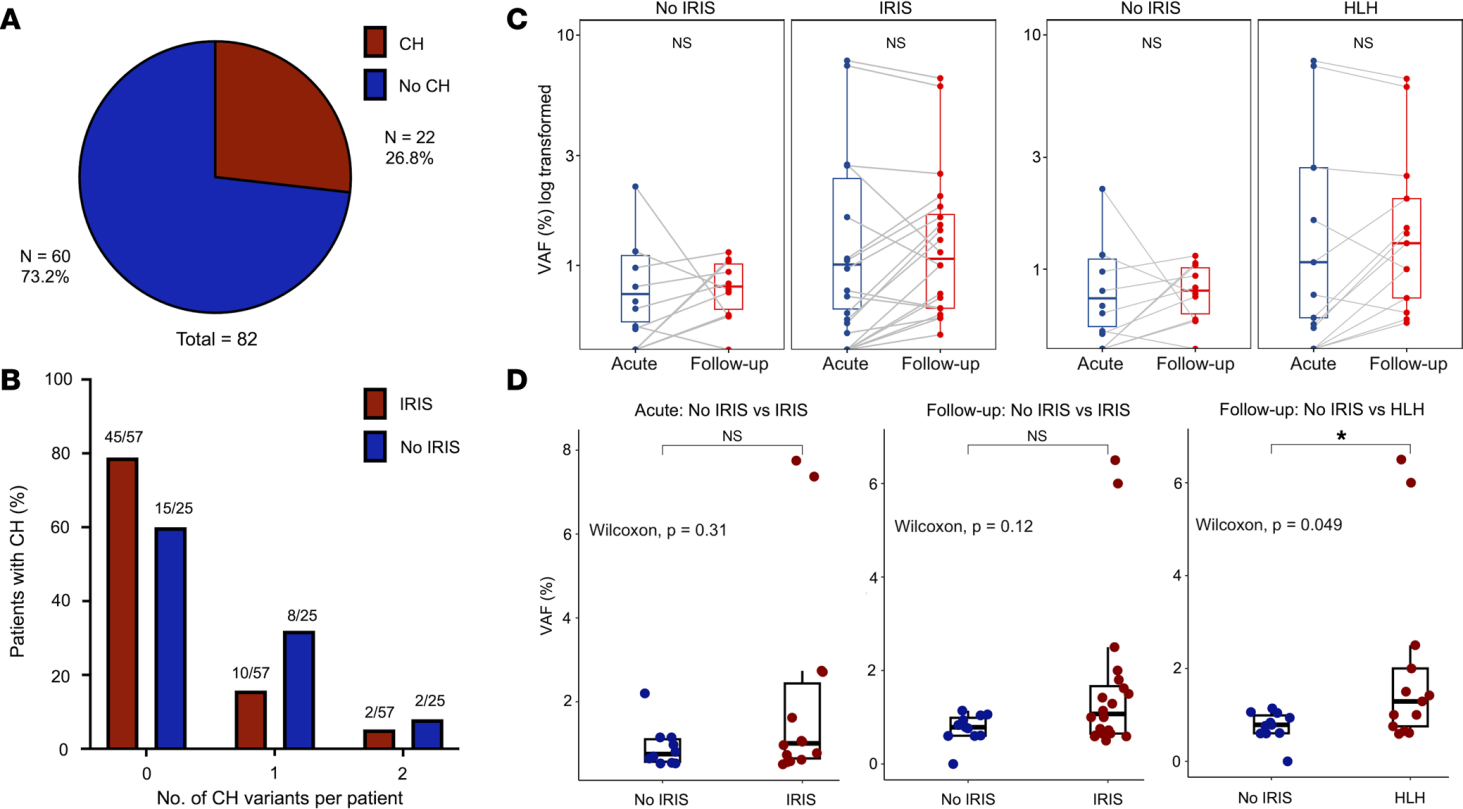
Characteristics of clonal hematopoiesis in people with HIV at initiation of antiretroviral therapy and longitudinal changes after immune reconstitution. (**A**) Proportion of participants with any clonal hematopoiesis (CH) mutation at the Acute time point (time of antiretroviral therapy initiation). (**B**) Number of CH variants identified at the Acute time point per individual stratified by development of immune reconstitution inflammatory syndrome (IRIS). (**C**) Longitudinal trend in variant allele frequency (VAF; log transformed) from Acute time point to Follow-up time point by inflammatory syndrome status (No IRIS, IRIS, HLH). HLH occurred in setting of, or prior to IRIS, in all individuals. Data are presented as box-and-whisker plots with medians (lines within boxes) and IQRs (box bounds). Groups were compared using pairwise Wilcoxon’s rank sum test. (**D**) Comparison of VAF between those without IRIS to those with IRIS with or without HLH at the Acute and Follow-up time points. Groups were compared using Wilcoxon’s rank sum test and resultant *P* values are shown. *P < 0.05.

Rates of CH have recently been shown to be independently higher in an adult cohort with secondary HLH caused by malignancies and infections after adjusting for key covariates (34). Mouse studies also reported that engineered CH could enhance the inflammatory phenotype in a murine model of secondary HLH (34). Therefore, in a subset of 82 PWH with mycobacterial and/or severe inflammatory complications, including IRIS and HLH, we evaluated for the presence and VAF of CH mutations at the initiation of ART to determine the potential impact of CH on the development of these inflammatory complications. Among PWH with Acute and Follow-up time point samples available, 74 (91.4%) had mycobacterial infections, 11 (13.6%) had KSHV-associated syndromes, and 3 (3.7%) had lymphoma. In this subset, 27 CH variants were identified in 22 PWH at the Acute time point at the time of ART initiation (Figure 4, A and B). The median VAF in the acute samples was 0.97% (IQR: 0.60–1.91). CH variants primarily occurred in *DNMT3A* (*n* = 10) and *TET2* (*n* = 7), with multiple variants also identified in *TP53* (*n* = 3) and *STAT3* (*n* = 2).

There were no statistically significant associations between presence of CH in the Acute time point samples and subsequent development of IRIS with or without HLH. However, 17 participants had the same CH variants identified at both the Acute and Follow-up time points (between 80 and 115 weeks after ART initiation), and 9 (52.9%) of these patients had severe IRIS with or without HLH, with 6 (35.3%) individuals requiring prolonged immunosuppression for over 6 months. PWH with IRIS with or without HLH had a greater increase in VAF between the Acute and Follow-up time points (median increase in VAF of 0.6%) compared with those without IRIS (median VAF increase of 0.16%), although this difference was not statistically significant (Figure 4, C and D). Notably, the comparison of VAFs between Acute and Follow-up unsorted PBMC samples can be affected by shifts in the percentage of peripheral blood monocytes versus T cells during immune reconstitution. Although the percentage of lymphocytes increased with treatment of HIV from the Acute to Follow-up time points, there were no significant changes in the monocyte percentages of PBMCs (Supplemental Figure 5), although even small shifts in cell subsets could potentially impact the VAF of CH populations.

## Discussion

PWH on ART remain at increased risk of CVD and serious non-AIDS adverse events, including non-AIDS malignancies despite virologic suppression (5–7). This is further compounded by aging, as it is estimated that greater than 70% of PWH by 2030 will be more than 50 years of age. Identifying mechanisms of immune dysregulation and risk stratification tools for CVD and other complications such as cancers in this population is a high priority. CH is associated with increased mortality due to a greater risk of CVD in older populations (15, 35). Early studies of CH prevalence in PWH have identified increased rates compared with non-HIV populations, providing a possible mechanism for the increased CVD and non-AIDS, nonhepatic cancer risk in PWH (25–29). These studies primarily focused on age-matched comparisons to non-HIV populations and included older adults with median ages over 50 years, whereas our cohort was younger (median age 42 years), with greater rates of prior advanced HIV and OIs.

We identified CH in 27.4% of patients with variants primarily identified in *DNMT3A* and *TET2*, consistent with prior cohorts of HIV and non-HIV populations (13, 25, 26, 35). Abelson et al. in 2018 used a similar targeted sequencing approach as our study, with a sensitivity to detect VAF of 0.5% (36). Although their gene panel was broader (101 genes), they found CH in 16% (24/146) of otherwise healthy individuals between the ages of 36 and 52 years. This prevalence was notably lower than the rates identified in our cohort of PWH, which remained at 24% even after matching for the genes evaluated in their study. In fact, CH was found in 13.8% of PWH less than 40 years of age in our cohort. These differences suggest the risk of CH and/or the rate of clonal expansion may be significantly greater in PWH even at younger ages, especially in those with a history of advanced HIV and OIs.

Comparing the CH prevalence in our HIV cohort to other published cohorts of PWH is limited by variability in sequencing methodology and cohort characteristics (25–29). Studies in PWH using whole-exome data, with a lower sensitivity to detect CH at a VAF of 2% or greater, have found prevalences between 4% and 14% (26–28). Using a 24-gene panel, a study by van der Heijden et al. found a prevalence of 21.2% in PWH, although this panel did not include *DNMT3A* (27). Dharan et al. used a similar sequencing methodology, with a 55-gene panel able to detect a VAF of 0.5%. They found a similar CH prevalence of 28.2%; however, their cohort was notably older, with a median age of 63 years, and only 34.6% had a CD4^+^ nadir of less than 200 cells/μL, with 17% having a prior AIDS-defining condition (25).

In our cohort, PWH and CH also demonstrated increased inflammatory biomarkers related to innate immune activation (sCD14, IL-8 — associated with CVD), suggesting greater systemic inflammation and immune dysregulation. Although other studies have shown increased IL-6 and C-reactive protein in PWH and CH compared with non-HIV populations (25), even people with well-controlled HIV are known to have increases in inflammatory biomarkers compared with age-matched populations (37). We expand on these findings by demonstrating increased levels of inflammatory biomarkers in PWH with CH compared with PWH without CH. There was a trend toward increased vWF as well, which together with higher levels of D-dimer was previously reported in another cohort in PWH with CH independent of age and nadir CD4^+^ T cells (29).

Our analysis attempted to identify HIV-specific clinical and laboratory risk factors associated with CH. In an ethnically and racially diverse cohort, where most individuals presented with advanced HIV (CD4^+^ < 200 cells/μL) and thus likely after extended periods of chronic inflammation and infections, current CD8^+^ T cell counts and nadir CD4^+^ T cell counts were the strongest factors associated with CH. Prior studies have also identified a correlation between nadir CD4^+^ and risk of CH (25, 29); however, they included fewer people with a nadir CD4^+^ of less than 200, compared with our cohort where over 75% of individuals had a history of advanced HIV and the median nadir CD4^+^ T cell count was 33 cells/μL. This is the first study to our knowledge to demonstrate an association between CD8^+^ T cell counts and CH in PWH. This finding remained significant in the multivariate model and was stronger than the association of the CD4^+^/CD8^+^ ratio. There was no relationship between CH and current CD4^+^ T cell count, similar to prior studies (29).

Increased CD8^+^ T cell counts are associated with serious non-AIDS adverse events and increased mortality in PWH, potentially providing another link between CH and poor long-term clinical outcomes (38, 39). Prior studies have shown that expanded CD8^+^ T cells in PWH have a more differentiated effector or senescent cell phenotype and they express higher markers of activation, potentially leading to chronic immune dysregulation (38–40). Individuals with this immunophenotype also demonstrate decreases in naive T cells even after control of HIV and immune reconstitution, which is similar to the immunophenotype found in individuals with increased inflammatory aging that also links an expansion of effector CD8^+^ T cells with greater vascular disease and mortality (41).

Notably, cell lineage analysis identified *DNMT3A* variants within T cells at even greater VAFs than in monocytes in several patients. Individuals with *DNMT3A* CH have been previously reported to more frequently carry CH mutations in T cells than CH involving other genes, potentially due to acquisition of these mutations in an earlier stem cell progenitor, or less myeloid bias in lineage output for *DNMT3A* mutations. In advanced HIV, especially in those with inflammatory complications, the depletion of CD4^+^ T cells during AIDS and chronic stimulation from persistent viremia leads to a dysregulated and exhausted T cell compartment (42). Since loss of *DNMT3A* in T cells is associated with retained proliferative and effector capacity in the setting of chronic viral infections (43, 44), it is possible this subpopulation has a survival advantage and is more capable of expansion after HIV is suppressed with ART.

Mechanistically, it is not clear whether advanced immunodeficiency is the major driver of more rapid clonal expansion, or whether CH occurs due to prolonged, persistent immune activation driven by HIV viremia and dysregulated T cell responses. There was a strong trend toward increased CH in those who developed inflammatory syndromes such as IRIS or the more severe inflammatory subset of individuals with HLH, many of whom had concomitant mycobacterial infections, suggesting these additional inflammatory insults may further increase CH risk and not the reverse. Murine models for CH have demonstrated that chronic mycobacterial infection can expand *DNMT3A*-mutant clones via increased IFN-γ signaling (19), and the pathogenesis of IRIS and HLH is heavily driven by pathologic IFN-γ production and an increase in activated T cells (22, 45). In fact, IRIS and CD8^+^ T cells were the only significant covariates in a logistic regression model specifically evaluating for *DNMT3A* variants. This association between inflammatory disease and CH is consistent with other models demonstrating that chronic infection and inflammation drive CH development and expansion (16, 19, 23, 24).

Longitudinally, there was persistence and possibly a trend toward an expansion of CH populations even over a relatively short follow-up of approximately 2 years. This increase was more prominent in individuals with severe, prolonged inflammatory syndromes. Under the stress of this hyperinflammatory state, HSCs and myeloid cells carrying CH mutations in genes such as *DNMT3A* and *TET2* may have a competitive advantage (16, 17, 19). PWH with IRIS with or without HLH were generally younger in our cohort. In these individuals with CH, despite suppression of HIV and treatment of underlying OIs, these clonal populations may lead to chronic immune dysregulation and significant risk for long-term adverse events. Therefore, this group represents an important population to monitor going forward, as they may require more aggressive CVD risk reduction and cancer screening with aging. Monitoring the dynamics of these CH populations will be vital to elucidate their evolution over time in the setting of well-controlled HIV even after control of their prior inflammatory syndromes.

We also evaluated whether CH at the time of ART initiation could increase the risk of IRIS or HLH. CH can enhance inflammatory disease pathology in murine models of HLH (34). Heterozygous germline variants in HLH-associated genes are enriched in PWH who develop mycobacterial IRIS (46), and we hypothesized that a similar mechanism could occur in the setting of CH due to the overlapping pathogenesis of mycobacterial IRIS and HLH (22). However, no significant difference was identified in CH between those who developed or did not develop IRIS. For patients with CH who did develop IRIS, the VAF was overall small (median VAF: 1.05%), making it unlikely that dysregulation in this small clonal monocyte population could dramatically impact inflammatory disease severity.

Our study has some limitations. The cohort was imbalanced, with an increased percentage of younger patients with prior advanced HIV and episodes of IRIS with or without HLH. To limit this confounding, we generated multivariate logistic regression models to control for age and other important confounders. Furthermore, since the relationship between age and CH is nonlinear, we also performed a sensitivity analysis, excluding the youngest quartile of patients to preserve power while evaluating an older group of patients who would be at higher risk of CH. A strength of our study was the inclusion of a wide diversity of patients in terms of age, sex, race/ethnicity, and clinical history; however, this also becomes a limitation due to the difficulty in controlling and evaluating for a large number of covariates. The cohort was very well characterized and followed longitudinally, which limits the risk of clinical misclassifications. The sample size was relatively small, and biomarker results were not available for every patient. Finally, since the NIH is a referral institution there is a possibility for sampling bias, as severely affected patients may be referred more frequently.

In conclusion, we provide the first description to our knowledge of CH prevalence and characteristics in a cohort with primarily advanced HIV and a diversity of OIs and inflammatory complications. Risk of CH was significantly associated with CD8^+^ T cell counts and nadir CD4^+^ T cell counts and may be further increased in those who develop inflammatory syndromes due to coinfections with mycobacteria and/or KSHV. Persistent immune dysregulation and activation in PWH associate with CH development and expansion consistent with other disease models. Further evaluation of the dynamic changes in these CH populations over time is essential, as this patient population may be at the highest risk of long-term serious non-AIDS adverse events, including CVD, stroke, and malignancies and may benefit from aggressive risk reduction strategies and possibly targeted immune modulators.

## Methods

### Sex as a biological variable.

This study included individuals of both male and female sexes. Sex was included as a variable in logistic regression models.

### Clinical cohort.

In total, 197 PWH were included in this study, all were previously enrolled in 1 of 3 prior National Institute of Allergy and Infectious Diseases (NIAID) IRB-approved observational, prospective studies of PWH (ClinicalTrials.gov NCT00286767, NCT02147405, and NCT02081638). Briefly, NCT00286767 and NCT02147405 were observational clinical trials in those with advanced HIV and CD4^+^ T cells of less than 100 cells/μL, whereas NCT02081638 recruited individuals with well-controlled HIV on ART as well as LTNP/EC (protocol details in Supplemental Methods). All participants were evaluated at the NIH Clinical Center. PBMCs were collected from all individuals with a suppressed HIV viral load for at least 1 year (Follow-up time point). No individuals were on corticosteroids or had untreated OIs at the time of this sample collection. A subset of 82 patients with mycobacterial infections and/or severe IRIS had PBMC samples available from before ART initiation or during the IRIS event and these samples were also evaluated for CH (Acute time point). Genomic DNA was isolated from PBMCs using a Gentra PureGene Cell kit (Qiagen).

IRIS events were defined using the AIDS Clinical Trials Group IRIS definition criteria (47). Individuals with severe IRIS and meeting clinical laboratory criteria for HLH and/or macrophage activation syndrome (MAS) were subclassified as an HLH subgroup. Since these criteria were applied retrospectively, all published HLH/MAS criteria were utilized (48–50) to broadly capture patients demonstrating an HLH/MAS clinical phenotype. All participants signed informed consent and all procedures were in accordance with the Declaration of Helsinki.

### Error-corrected deep sequencing for CH.

Error-corrected sequencing for CH was performed using a standard 56-gene panel (SureSelect, Agilent, ELID 3156971; Supplemental Table 3) as previously described (31). Briefly, 200 ng of genomic DNA was sheared to 250-bp fragments, and end repair, A-tailing, and adaptor ligation were performed with Molecular Index Adaptor and Random Molecular Barcodes to allow for error correction and detection of ultra-low-frequency variants. Hybridization-based enrichment and final library construction were performed according to Agilent’s SureSelect protocol. Libraries were sequenced on an Illumina NovaSeq 6000 system.

### Driver CH variant identification.

Variants were identified using an established protocol (51). Briefly, all sequences were aligned to the hg38 version of the reference human genome using bwa 0.7.17 in alt contig aware mode, as described previously (52). The generated SAM file was compressed into a BAM file and sorted by genomic position using samtools 1.9 (53). Error correction was done by running locatIT (version 2.0.2, Agilent) with default parameters, permitting 1 base mismatch within each molecular barcode. Consensus families comprising single reads were removed. PCR duplicates were marked using Picard’s MarkDuplicates (version 2.17.0, http://broadinstitute.github.io/picard/). SNPs and indels were called using Shearwater (version 3.15) (54, 55) and GATK’s Mutect2 (version 4.0.3.0) (56), respectively, following best practices. Reads with mapping quality below 30 were removed by Shearwater and below 25 by Mutect2 filtering steps. Nonsynonymous coding variants were filtered for (a) minimal 4 reads in each direction, (b) minimal VAF of 0.5%, (c) maximum VAF of 40%, and (d) a minor allele frequency in the SNP databases (1000 Genomes global minor allele frequency, gnomAD [exomes] allele frequencies) less than 0.1 % unless with a COSMIC occurrence of 100 or more. Driver CH variants were further identified according to the established criteria (Supplemental Table 4).

### Mutation validation and quantification in lineage-purified cells.

Targeted deep sequencing was used to validate CH variants identified by the CH panel sequencing and contributions to various blood lineages. CD4^+^ T cells, CD8^+^ T cells, and monocytes were each isolated by magnetic bead negative selection using respective EasySep isolation kits (STEMCELL Technologies). Genomic DNA was isolated from separated cell populations using the Gentra PureGene Cell kit (Qiagen). Targeted sequencing libraries were constructed via a 2-step PCR strategy (57). Briefly, the first round of PCR amplification was done using target-specific primers with adaptors followed by a second round of PCR amplification with NEBNext Multiplex Oligos for Illumina (Dual Index Primers Set 1; New England Biolabs). Gene-specific primers were designed using Primer3Plus (https://primer3.ut.ee/). Amplicons were approximately 100–150 bp. Forward and reverse adaptors for the NEBNext Multiplex were added 5′ of the gene-specific primers. The target-specific primers and adaptor sequences are provided in Supplemental Table 4. For the first round of PCR, 6.25 μL of KAPA HiFi HotStart ReadyMix polymerase (KAPA Biosystems) and 0.375 μL of each 10 μM primer were added to 25 ng of cellular DNA. For the second round of PCR, a unique combination of 0.75 μL of i5 and i7 primers from the NEBNext kit were added, plus an additional 11 μL of the KAPA polymerase. Cycling conditions for each PCR were 3 minutes at 95°C; 14 cycles of 98°C for 20 seconds, 62°C for 15 seconds, and 72°C for 30 seconds; followed by 1 minute at 72°C. Sequencing was performed on an Illumina MiSeq system with 150-bp paired-end reads at a depth of generally greater than 20,000× reads. Sequencing alignments were done similarly to the CH screening. Variant calling was done with freeBayes (58). Read counts and VAFs from known mutated positions were extracted and manually verified in IGV (59).

### Statistics.

Continuous variables are represented by median values with IQR and categorical variables by frequencies. Logistic regression modeling was performed to determine the impact of key covariates while controlling for known cofactors associated with CH, including age, sex, race, tobacco use, and history of lymphoma and/or chemotherapy. Significant covariates were fed into a multivariate stepwise selection process to form a multivariate model that also included the previously mentioned covariates. Plasma biomarker levels measured and previously reported in this cohort of PWH (22, 33) were reanalyzed in the context of the presence or absence of CH. Statistical comparisons were made using Wilcoxon’s rank sum test. A *P* value of less than 0.05 was considered statistically significant. All analyses were conducted using R software (version 4.3.2).

### Study approval.

The patients and healthy donors were enrolled in protocols approved by the NIH IRB and provided written informed consent for participation in the study. This study was conducted in accordance with the Declaration of Helsinki. All participants were enrolled in at least 1 of 3 observational, prospective clinical protocols (ClinicalTrials.gov NCT00286767, NCT02147405, and NCT02081638).

### Data availability.

For access to the original data and analytical code, requests should be directed to the corresponding and co–corresponding authors. Supporting Data Values are provided in an Excel file for each figure.

## Author contributions

JMR and YZ are co–first authors, listed alphabetically. JMR, YZ, NSL, AR, SLL, AMOV, SY, GSV, CED, and IS performed experiments and analyzed the data. JMR, YZ, NSL, SLL, SY, GSV, CED, and IS designed experiments. JMR, EL, FG, MVA, AL, MM, and IS provided clinical care and referred patients. FG and MVA provided regulatory support to patient enrollment and consenting. CED and IS conceived and supervised the project. JMR and YZ wrote the final manuscript. NSL, EL, FG, MVA, AR, SLL, AMOV, AL, MM, GSV, CED, and IS reviewed and revised the manuscript.

## Supplementary Material

Supplemental data

Supporting data values

## Figures and Tables

**Table 1:**
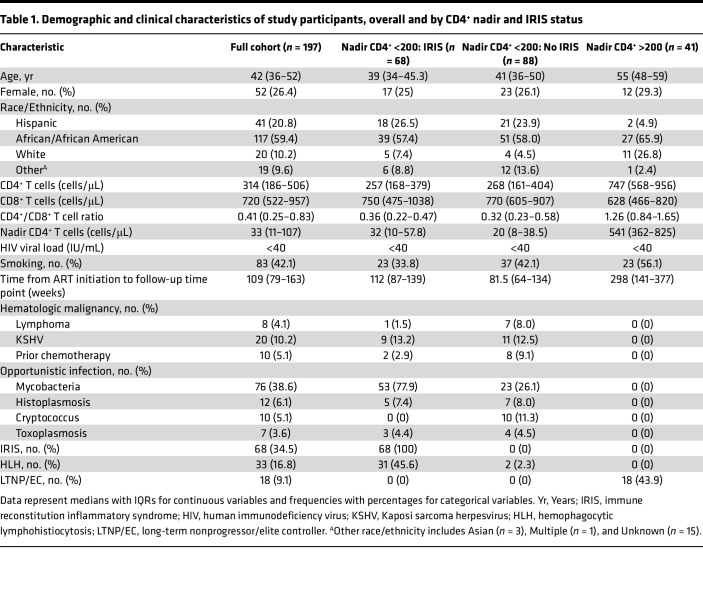
Demographic and clinical characteristics of study participants, overall and by CD4^+^ nadir and IRIS status

## Version 1 04/02/2024

In-Press Preview

## Version 2 05/08/2024

Electronic publication
