# Supplementary material for: Clonal hematopoiesis in people with advanced HIV and associated inflammatory syndromes
Source: JCI Insight. 2024 Apr 2;9(9):e174783. doi: 10.1172/jci.insight.174783 (PMC11141903; doi:10.1172/jci.insight.174783)
Supplement: Supplemental data [file jciinsight-9-174783-s006.pdf]

## Supplemental Methods

### Clinical Protocols

All 197-participants were previously enrolled in one of three prior National Institute of Allergy and Infectious Diseases institutional review board approved observational, prospective studies of individuals with HIV (NCT00286767, NCT02147405, NCT02081638). These included the "Immune Reconstitution Inflammatory Syndrome in HIV-Infected Patients Taking Antiretroviral Therapy" study (IRIS - NCT00286767) and the "PET Imaging and Lymph Node Assessment of IRIS in Persons with AIDS" study (PANDORA - NCT02147405) which were both observational longitudinal studies evaluating the immunopathogenesis of IRIS in HIV-1 infected patients with CD4 count <100 cells/μL. All patients were seen and evaluated at the National Institutes of Health in Maryland, USA. In both studies, IRIS events were defined using the AIDS Clinical Trials Group IRIS definition criteria which include evidence of ART initiation with resultant increase in CD4 count (≥50 cells/μL or a ≥2-fold rise) and/or virologic suppression (>0.5 log10 decrease in plasma HIV viremia), clinical presentation consistent with an infectious or inflammatory condition, and the absence of an alternative etiology such as the expected course of a previously recognized infection or side-effects of medications. Peripheral blood mononuclear cells (PBMCs) were collected prior to ART and at weeks 2, 4, 8, 12, 24, 36, 48, 64, 80, 96 and during episodes of IRIS. The third study (NCT02081638) was the "Elite Controller and ART-treated HIV+ Statin Versus ASA Treatment Intervention Study" (ECSTATIN) which was an open label randomized controlled trial of either aspirin or atorvastatin given for 9-months to study their potential effect in immune activation and inflammation. All participants were 18 years or older with plasma viremia <40 copies/ml. Women who were lactating or were pregnant, or actively trying to become pregnant were excluded from participation. Other exclusion criteria

were known hypersensitivity or allergy or other contraindication to study medications, diagnosis of cardiovascular disease or hypercholesterolemia (LDL cholesterol ≥ 190 mg/dL), any chronic inflammatory condition requiring treatment with anti-inflammatory medications, active drug or alcohol use disorder. All participants signed informed consent and all procedures were in accordance with the Helsinki Declaration of the World Medical Association.

## Supplemental Figures

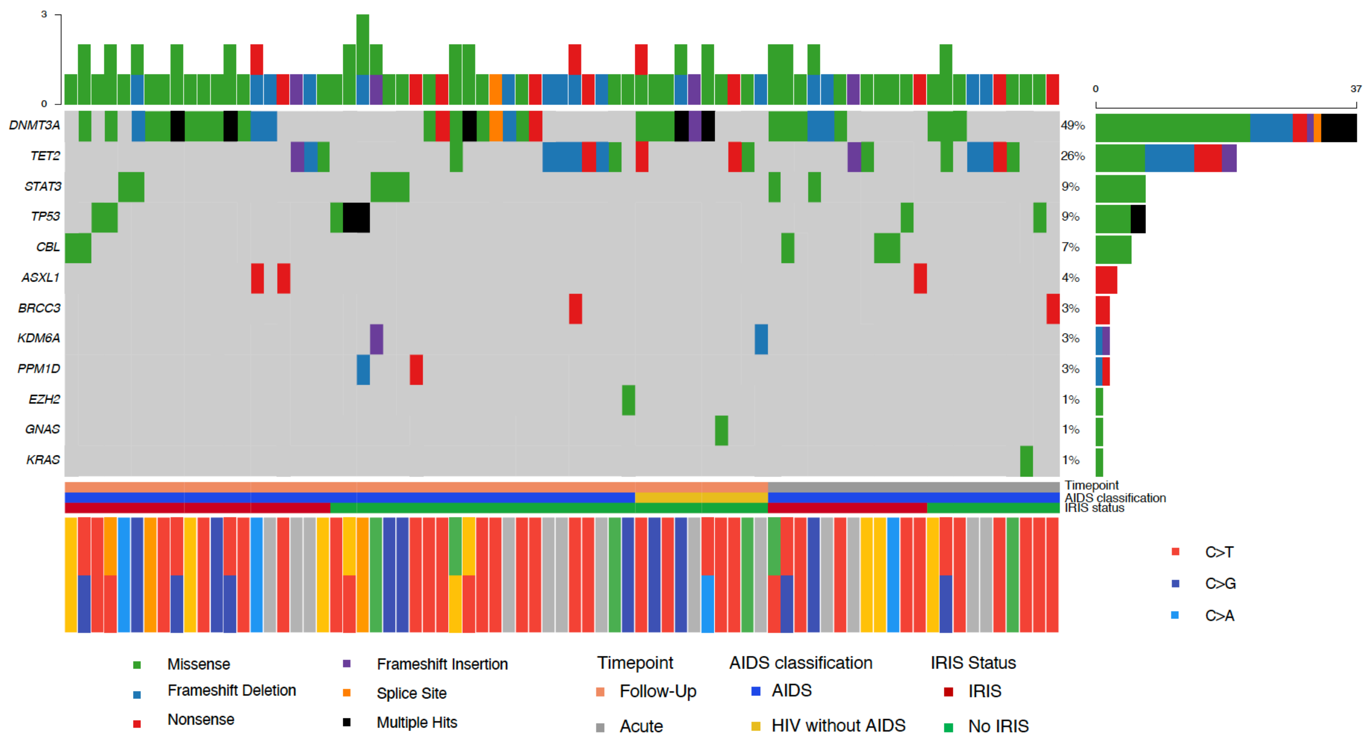


**Supplemental Figure 1:** Waterfall plot demonstrating specific clonal hematopoiesis variants in each gene for individual patients at the acute and follow-up timepoints. All gene mutations (N=100) in 54-people with HIV are depicted, and the proportion of each specific mutation identified is depicted on the right. Subgroups at the bottom represent the Acute and Follow-up Timepoints, history of CD4 nadir <200 cells/μL (AIDS classification), and history of immune reconstitution inflammatory syndrome (IRIS status). Specific nucleotide changes are also color-coded and summarized at the bottom of the figure.

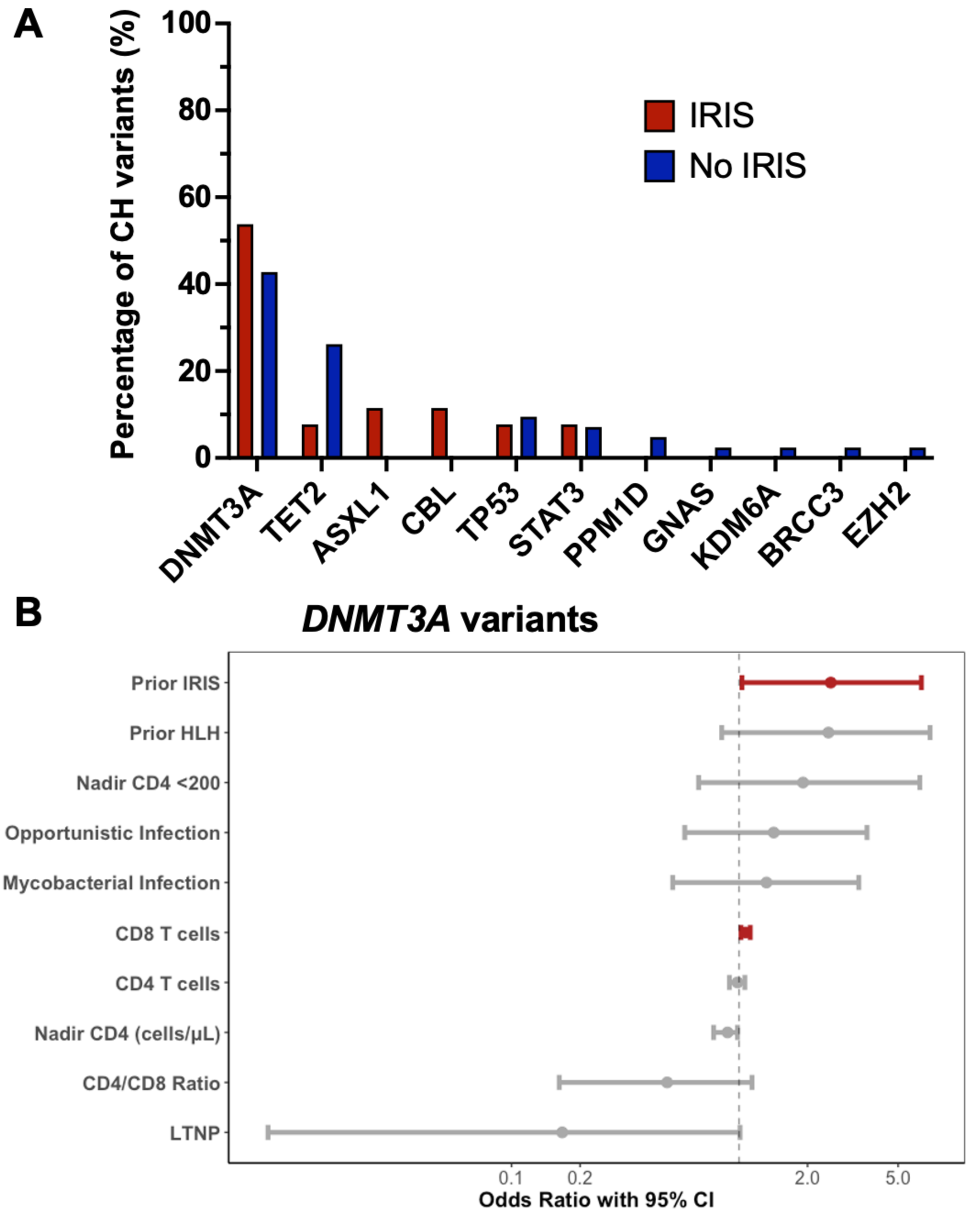


**Supplemental Figure 2:** (A) Proportion of clonal hematopoiesis variants identified in each gene at the follow-up timepoint stratified by history of IRIS. (B) Adjusted logistic regression with 95% CIs of key variables and their impact on risk of clonal hematopoiesis in *DNMT3A* in people with HIV. CD4 and CD8 T-cell counts are presented as an OR per 50 cells/μL change. CD4 T-cell nadir was evaluated as a categorical variable (CD4 T-cells nadir <200 cells/μL) and a continuous variable (per 50 cells/μL change).

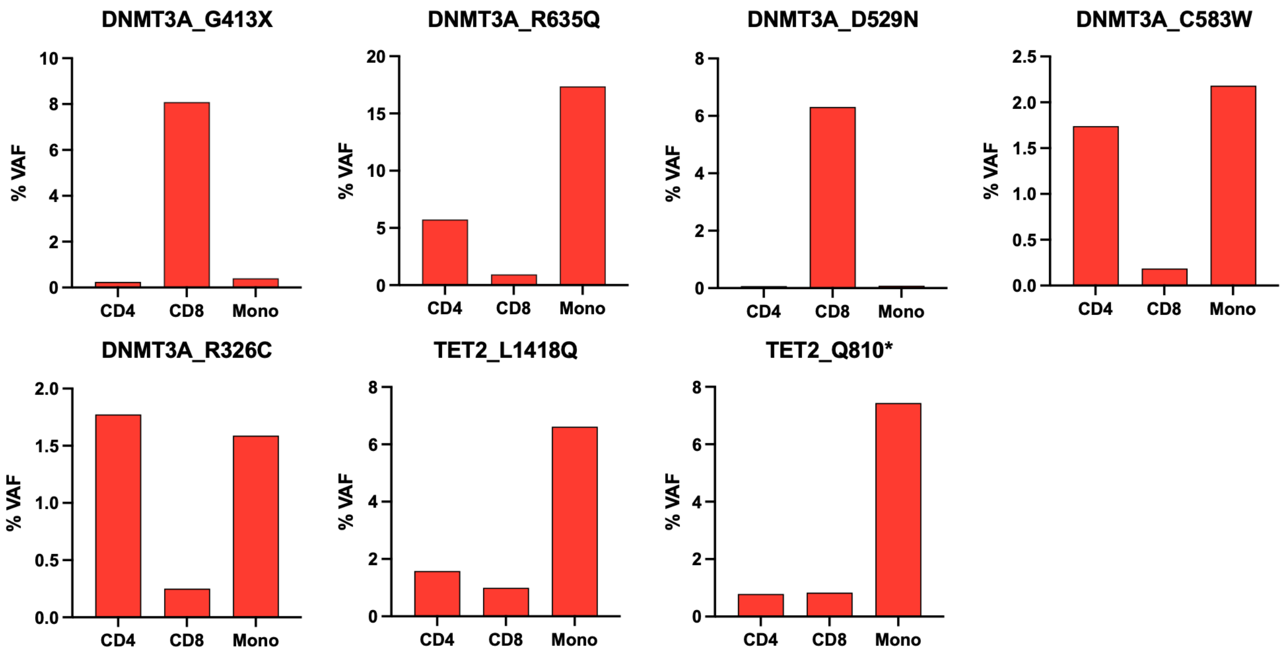


**Supplemental Figure 3:** Variant allele frequencies in purified peripheral blood lineages of 7-participants with HIV and clonal hematopoiesis in *DNMT3A* or *TET2*. Abbreviations: VAF – variant allele frequency; Mono – monocytes; CD4 – CD4 T-cells; CD8 – CD8 T-cells.

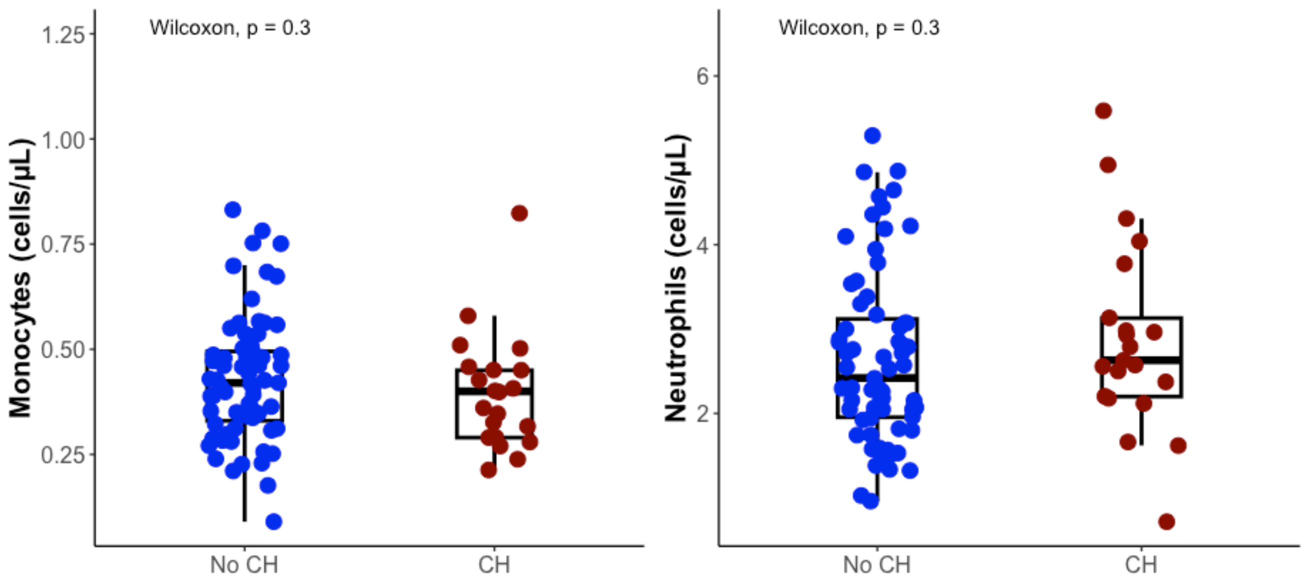


**Supplemental Figure 4:** Comparison of peripheral blood absolute monocyte and neutrophil counts between the people with HIV without clonal hematopoiesis and with clonal hematopoiesis. Data are presented as boxplots with medians and interquartile ranges. Groups were compared using Wilcoxon rank sum test and resultant p-values are shown.

Abbreviations: CH - clonal hematopoiesis.

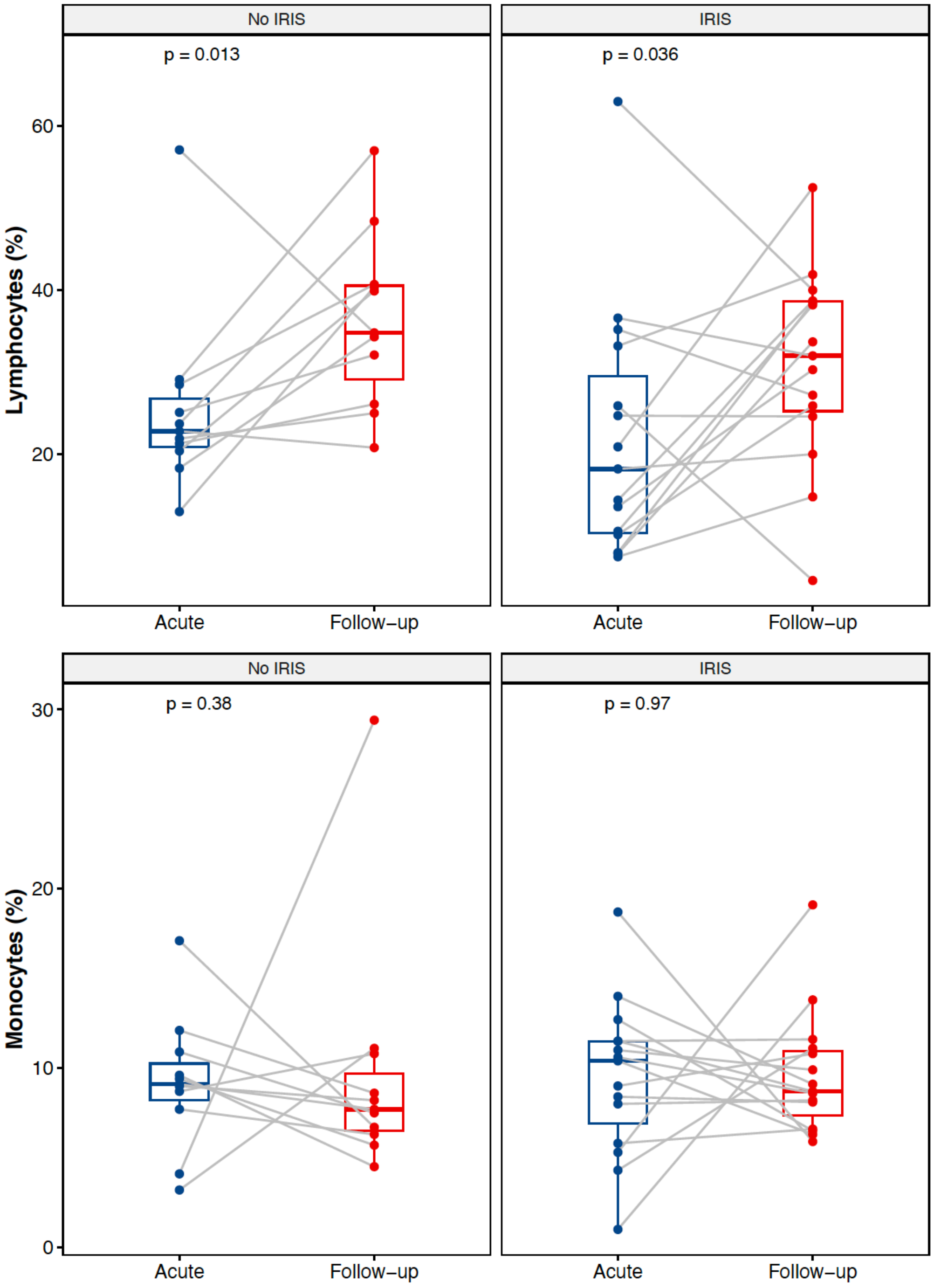


**Supplemental Figure 5:** Comparison of peripheral blood monocyte and lymphocyte frequencies between the acute and follow-up sample in people with HIV and CH. Data are presented as boxplots with medians and interquartile ranges. Groups were compared using Wilcoxon rank sum test and resultant p-values are shown.

**Supplemental Tables**

**Supplemental Table 1:** Demographic and clinical characteristics of people with HIV with or without clonal hematopoiesis.

| **Characteristic** | **CH** (n = 54) | **No CH** (n = 143) |
|---|---|---|
| Age (yr) | 48 (41-55) | 40 (35-50) |
| Female – no. (%) | 14 (26) | 38 (27) |
| **Race/Ethnicity – no. (%)** | | |
| Hispanic | 5 (9.3) | 38 (26.6) |
| African/African-American | 34 (63) | 78 (54.5) |
| White | 9 (16.6) | 13 (9.1) |
| Other[A] | 6 (11.1) | 14 (9.8) |
| Nadir CD4 T-cells (cells/μL) | 36 (11-103) | 32 (11-107) |
| HIV viral load (IU/mL) | <40 | <40 |
| Smoking – no. (%) | 25 (46) | 58 (41) |
| **Hematologic malignancy – no. (%)** | | |
| Lymphoma | 5 (9.3) | 3 (2.1) |

| | | |
|---|---|---|
| KSHV | 2 (3.7) | 18 (12.6) |
| Prior Chemotherapy | 3 (5.6) | 7 (4.9) |
| **Opportunistic Infection – no. (%)** | | |
| Mycobacteria | 22(40.7) | 53 (37.1) |
| Histoplasmosis | 3 (5.6) | 9 (6.3) |
| Cryptococcus | 2 (3.7) | 8 (5.6) |
| Toxoplasmosis | 2 (3.7) | 5 (3.5) |
| IRIS – no. (%) | 21 (38.9) | 47 (32.9) |
| HLH – no. (%) | 11 (20.4) | 22 (15.3) |
| LTNP – no. (%) | 4 (7.4) | 14 (9.8) |

Data represent medians with interquartile ranges for continuous variables and frequencies with percentages for categorical variables. Abbreviations: Yr – Years; IRIS – immune reconstitution inflammatory syndrome; HIV – human immunodeficiency virus; KSHV – Kaposi sarcoma herpesvirus; HLH – hemophagocytic lymphohistiocytosis; LTNP – long-term nonprogressor. [A]Other race/ethnicity includes: Asian (n=3), Multiple (n=1), and Unknown (n=15).

**Supplemental Table 2:** Primer and adapter sequences used for mutation validation

| | |
|---|---|
| DNMT3A_G413X_ 129bp Forward (adapter) | 5' ACACTCTTTCCCTACACGACGCTCTTCCGATCTACAGCGATG AGAGTGACACT 3' |
| DNMT3A_G413X_ 129bp Reverse (adapter) | 5' GACTGGAGTTCAGACGTGTGCTCTTCCGATCTGGTGCCCTCA TTTACCTTCTG 3' |
| DNMT3A_R635Q_ 145bp Forward (adapter) | 5' ACACTCTTTCCCTACACGACGCTCTTCCGATCTGTCCCAGCTG AGAAGAGGAA 3' |
| DNMT3A_R635Q_ 145bp Reverse (adapter) | 5' GACTGGAGTTCAGACGTGTGCTCTTCCGATCTTTCGTTTTGCC AGAGTTGCC 3' |
| DNMT3A_D529N_ 150bp Forward (adapter) | 5' ACACTCTTTCCCTACACGACGCTCTTCCGATCTCTGCTTTCTG GAGTGTGCG 3' |
| DNMT3A_D529N_ 150bp Reverse (adapter) | 5' GACTGGAGTTCAGACGTGTGCTCTTCCGATCTCCCAGCTAAG GAGACCACTG 3' |

| | |
|---|---|
| DNMT3A_C583W_149bp Forward (adapter) | 5' ACACTCTTTCCCTACACGACGCTCTTCCGATCTGTGGAGTGTGTGGACCTCTT3' |
| DNMT3A_C583W_149bp Reverse (adapter) | 5' GACTGGAGTTCAGACGTGTGCTCTTCCGATCTAAGAACATCTGGAGCCGGG3' |
| DNMT3A_R326C_150bp Forward (adapter) | 5' ACACTCTTTCCCTACACGACGCTCTTCCGATCTCTTTGGCATTGGGGAGCTG 3' |
| DNMT3A_R326C_150bp Reverse (adapter) | 5' GACTGGAGTTCAGACGTGTGCTCTTCCGATCTACCACTGAGAATTTGCCGTC 3' |
| TET2_Q810X_143bp Forward (adapter) | 5' ACACTCTTTCCCTACACGACGCTCTTCCGATCTTGGCCAGACTAAAGTGGAAGA 3' |
| TET2_Q810X_143bp Reverse (adapter) | 5' GACTGGAGTTCAGACGTGTGCTCTTCCGATCTTCATGGTCTGACTATAAGGGGA 3' |

| | |
|---|---|
| TET2_L1418Q_114bp Forward (adapter) | 5' ACACTCTTTCCCTACACGACGCTCTTCCGATCTACCAGGTATGCACTCTCACT 3' |
| TET2_L1418Q_114bp Reverse (adapter) | 5' GACTGGAGTTCAGACGTGTGCTCTTCCGATCTTCCCAAACTCATCCACGTCA 3' |
| TET2_I1873T_150bp Forward (adapter) | 5' ACACTCTTTCCCTACACGACGCTCTTCCGATCTCAGAGGACAACGATGAGGTC 3' |
| TET2_I1873T_150bp Forward (adapter) | 5' GACTGGAGTTCAGACGTGTGCTCTTCCGATCTTGGGATTCTTTAAAGGGGTTGTG 3' |

**Supplemental Table 3:** Target genes analyzed via panel targeted error-corrected sequencing

| | | | |
|---|---|---|---|
| *ASXL1* | *ETV6* | *MPL* | *SF1* |
| *BCOR* | *EZH2* | *MYD88* | *SF3A1* |
| *BCORL1* | *FLT3* | *NOTCH1* | *SF3B1* |
| *BRAF* | *GATA2* | *NPM1* | *SMC1A* |
| *BRCC3* | *GNAS* | *NRAS* | *SMC3* |
| *CALR* | *GNB1* | *PHF6* | *SRSF2* |
| *CBL* | *IDH1* | *PIGA* | *STAG2* |
| *CEBPA* | *IDH2* | *PPM1D* | *STAT3* |
| *CREBBP* | *JAK2* | *PRPF40B* | *TET2* |
| *CSF1R* | *JAK3* | *PTEN* | *TP53* |
| *CSF3R* | *KDM6A* | *PTPN11* | *U2AF1* |
| *CTCF* | *KIT* | *RAD21* | *U2AF2* |
| *CUX1* | *KMT2A* | *RUNX1* | *WT1* |
| *DNMT3A* | *KRAS* | *SETBP1* | *ZRSR2* |

**Supplemental Table 4:** Clonal Hematopoiesis Driver classification criteria

| Gene name | Criteria for classification as a driver mutation | Transcript |
|---|---|---|
| ASXL1 | Frameshift/nonsense/splice-site in exon 11-12 | NM_015338 |
| BCOR | Frameshift/nonsense/splice-site | NM_001123385 |
| BCORL1 | Frameshift/nonsense/splice-site | NM_021946 |
| BRAF | Missense in aa range p.590-615; Missense at G469 | NM_004333 |
| BRCC3 | Frameshift/nonsense/splice-site | NM_024332 |
| CALR | Frameshift in exon 9 | NM_004343 |
| CBL | Missense in Linker/RING finger domains (p.345-434) | NM_005188 |
| CEBPA | Frameshift/nonsense/splice-site | NM_004364 |

| | | |
|---|---|---|
| CREBBP | Frameshift/nonsense/splice-site | NM_004380 |
| CSF1R | Missense at L301 / Y969 | NM_005211 |
| CSF3R | T615A, T618I, truncating c.741-791 | NM_000760 |
| CTCF | Frameshift/nonsense/splice-site, R377C, R377H, P378A, P378L | NM_006565 |
| CUX1 | Frameshift/nonsense/splice-site | NM_181552 |
| DNMT3A | Frameshift/nonsense/splice-site; Missense in PWWP (p.292-350) / ADD (p.482-614) / MTase (p.634-912) domains | NM_022552 |
| ETV6 | Frameshift/nonsense/splice-site | NM_001987 |
| EZH2 | Frameshift/nonsense/splice-site; Missense in SET domain (p.617-732) | NM_001203247 |
| FLT3 | V579A, V592A, V592I, F594L, FY590-591GD, D835Y, D835H, D835E, del835 | NM_004119 |
| GATA2 | Frameshift/nonsense/splice-site, R293Q, N317H, A318T, A318V, A318G, G320D, L321P, L321F, L321V, Q328P, R330Q, R361L, L359V, A372T, R384G, R384K | NM_001145661 |
| GNAS | Missense at R201 (844) | NM_016592 |
| GNB1 | Missense at K57 / I80 | NM_002074 |
| IDH1 | Missense at R132 | NM_005896 |
| IDH2 | Missense at R140 / R172 | NM_002168 |
| JAK2 | V617F; Missense/indel in aa range p.536-547 | NM_004972 |
| JAK3 | M511T, M511I, A572V, A572T, A573V, R657Q, V715I, V715A | NM_000215 |

| | | |
|---|---|---|
| KDM6A | Frameshift/nonsense/splice-site | NM_021140 |
| KIT | ins503, V559A, V559D, V559G, V559I, V560D, V560A, V560G, V560E, del560, E561K, del579, P627L, P627T, R634W, K642E, K642Q, V654A, V654E, H697Y, H697D, E761D, K807R, D816H, D816Y, D816F, D816I, D816V, D816H, del551-559 | NM_000222 |
| KMT2A | Frameshift/nonsense/splice-site | NM_005933 |
| KRAS | Missense at G12 / G13 / Q61 / A146 | NM_033360 |
| MPL | S505G, S505N, S505C, L510P, del513, W515A, W515R, W515K, W515S, W515L, A519T, A519V, Y591D, W515-518KT | NM_005373 |
| MYD88 | L265P | NM_002468 |
| NOTCH1 | Frameshift/nonsense/splice-site/missense in exon 26-34 | NM_017617 |
| NPM1 | Frameshift in exon 12 | NM_002520 |
| NRAS | Missense at G12 / G13 / Q61 | NM_002524 |
| PHF6 | Frameshift/nonsense/splice-site | NM_001015877 |
| PIGA | Frameshift/nonsense/splice-site | NM_002641 |
| PPM1D | Frameshift/nonsense/splice-site in exon 5/6 | NM_003620 |
| PRPF40B | Frameshift/nonsense/splice-site | NM_001031698 |
| PTEN | Frameshift/nonsense/splice-site | NM_000314 |
| PTPN11 | Missense in aa range p.58-76 and p.491-510 | NM_002834 |
| RAD21 | Frameshift/nonsense/splice-site | NM_006265 |
| RUNX1 | Frameshift/nonsense/splice-site, S73F, H78Q, H78L, R80C, R80P, R80H, L85Q, P86L, P86H, S114L, D133Y, L134P, R135G, R135K, | NM_001001890 |

| | | |
|---|---|---|
| | R135S, R139Q, R142S, A165V, R174Q, R177L, R177Q, A224T, D171G, D171V, D171N, R205W, R223C | |
| SETBP1 | D868N, D868T, S869N, G870S, I871T, D880N, D880Q | NM_015559 |
| SF1 | Frameshift/nonsense/splice-site | NM_004630 |
| SF3A1 | Frameshift/nonsense/splice-site | NM_005877 |
| SF3B1 | Missense in terminal HEAT domains (p.529-1201) | NM_012433 |
| SMC1A | Missense at R96 / R586 | NM_006306 |
| SMC3 | Frameshift/nonsense/splice-site | NM_005445 |
| SRSF2 | Missense/deletion involving p.P95 | NM_003016 |
| STAG2 | Frameshift/nonsense/splice-site | NM_006603 |
| STAT3 | Missense in SH2 domain (p.580-670) | NM_139276 |
| TET2 | Frameshift/nonsense/splice-site; Missense in conserved domains (p.1104-1481 and p.1843-2002) | NM_001127208 |
| TP53 | Frameshift/nonsense/splice-site; Missense in DNA-binding domain (p.95-288); Missense at P72 / R337 | NM_001126112 |
| U2AF1 | Missense at S34 / R156 / Q157 | NM_006758 |
| U2AF2 | Missense in RNA recognition motifs domains (p.149-231, p.259-337, p.381-462) | NM_007279 |
| WT1 | Frameshift/nonsense/splice-site | NM_024426 |
| ZRSR2 | Frameshift/nonsense/splice-site | NM_005089 |
